# Translational Research for Tuberculosis Elimination: Priorities, Challenges, and Actions

**DOI:** 10.1371/journal.pmed.1001965

**Published:** 2016-03-02

**Authors:** Christian Lienhardt, Knut Lönnroth, Dick Menzies, Manica Balasegaram, Jeremiah Chakaya, Frank Cobelens, Jennifer Cohn, Claudia M. Denkinger, Thomas G. Evans, Gunilla Källenius, Gilla Kaplan, Ajay M. V. Kumar, Line Matthiessen, Charles S. Mgone, Valerie Mizrahi, Ya-diul Mukadi, Viet Nhung Nguyen, Anders Nordström, Christine F. Sizemore, Melvin Spigelman, S. Bertel Squire, Soumya Swaminathan, Paul D. Van Helden, Alimuddin Zumla, Karin Weyer, Diana Weil, Mario Raviglione

**Affiliations:** 1 World Health Organisation, Geneva, Switzerland; 2 Montreal Chest Institute, McGill University, Montreal, Canada; 3 Access Campaign, Médecins Sans Frontières, Geneva, Switzerland; 4 Centre for Respiratory Diseases Research, Kenya Medical Research Institute (KEMRI), Nairobi, Kenya; 5 Academic Medical Center, University of Amsterdam, Amsterdam, Netherlands; 6 FIND Diagnostics, Geneva, Switzerland; 7 Aeras, Rockville, Maryland, United States of America; 8 Department of Clinical Science and Education, Karolinska Institutet, Stockholm, Sweden; 9 Bill & Melinda Gates Foundation, Seattle, Washington, United States of America; 10 International Union Against Tuberculosis and Lung Disease, South-East Asia Regional Office, New Delhi, India; 11 DG Research & Innovation, European Commission, Brussels, Belgium; 12 The European & Developing Countries Clinical Trials Partnership (EDCTP), The Hague, The Netherlands; 13 Institute of Infectious Disease & Molecular Medicine, University of Cape Town, Cape Town, South Africa; 14 USAID, Washington, D.C., United States of America; 15 National TB Programme, National Lung Hospital, Hanoi, Viet Nam; 16 Ambassador for Global Health, Ministry for Foreign Affairs, Stockholm, Sweden; 17 Tuberculosis, Leprosy and other Mycobacterial Diseases Program, National Institute of Allergy and Infectious Diseases, National Institutes of Health, Rockville, Maryland, United States of America; 18 Global Alliance for TB Drug Development, New York, New York, United States of America; 19 Centre for Applied Health Research & Delivery, Liverpool School of Tropical Medicine, Liverpool, United Kingdom; 20 National Institute for Research in Tuberculosis (NIRT), Indian Council of Medical Research, Chennai, India; 21 DST/NRF Centre of Excellence for Biomedical Tuberculosis Research, MRC Centre for TB Research, Division of Molecular Biology and Human Genetics, Faculty of Medicine and Health Sciences, Stellenbosch University, Tygerberg, South Africa; 22 Division of Infection and Immunity, University College London Medical School, London, United Kingdom

## Abstract

Christian Lienhardt and colleagues describe the research efforts needed to end the global tuberculosis epidemic by 2035.

**Summary Points**

- The WHO End TB Strategy, endorsed by the World Health Assembly in May 2014, has the ambitious goal of ending the global tuberculosis (TB) epidemic by 2035, with targets of a 95% decline in deaths due to TB (compared with 2015) and a 90% reduction in incidence of TB to ten cases/100,000 or less and no TB-affected household experiencing catastrophic costs due to TB.
- Achieving this goal will only be possible through the development and rapid uptake of new tools, including rapid point-of-care diagnostics, safe and shorter treatment of latent TB infection and disease, and an efficacious TB vaccine, combined with efficient health systems and care provision, and actions on the social determinants of TB.
- Research for TB elimination requires an intensification of efforts across a continuum from fundamental research to clinical, epidemiological, implementation, health system, and social science research.
- Enhancing research along the full spectrum, from basic to implementation, and strengthening research capacity, particularly in low- and middle-income countries severely affected by the TB epidemics, is crucial for TB elimination.
- The creation of a research-enabling environment that fosters and rewards high-quality research requires a broad-based, concerted effort by national governments and international donors to develop and promote TB research and research capacity at the country level and the effective engagement of all stakeholders.

## Introduction

The WHO End TB Strategy, endorsed by the World Health Assembly in May 2014, has the ambitious goal of ending the global tuberculosis (TB) epidemic by 2035, with targets of a 95% decline in deaths due to TB (compared with 2015), a 90% reduction in incidence of TB to ten cases/100,000 or less, and no TB-affected household experiencing catastrophic costs due to TB [1]. To achieve these targets, a steep acceleration of the annual decline in global TB incidence will be required, from an average of 2% per year in 2015 to 10% per year by 2025. Such a decline was observed in Western European and North American countries after World War II within the context of expanded access to health care and rapid socioeconomic development [2]. While these measures, combined with optimal use of current tools and strategies, can produce up to a 10% reduction in incidence and deaths by 2025, a further acceleration of the decline in TB incidence to around 17% per year on average will be necessary to achieve the 2035 goals of the End TB Strategy. This acceleration will only be possible through the development and rapid uptake of new tools, including an efficacious TB vaccine, safe and shorter treatment of latent TB infection and disease, and rapid point-of-care diagnostics, combined with efficient health systems and care provision. Therefore, intensified research and innovation is one of the three fundamental pillars of the new WHO End TB Strategy (Box 1). Of note, these targets are consistent with the 2035 “convergence targets” proposed by the Commission on Investing in Health in its Global Health 2035 report [3]: the commission’s modelling suggested that a 91% reduction in TB deaths would be feasible in low-income and lower-middle-income countries from 2011 to 2035.

**Box 1. Components of the WHO END TB Strategy**

1. Integrated, Patient-Centred Care and Prevention
  A Early diagnosis of TB, including universal drug-susceptibility testing, and systematic screening of contacts and high-risk groups
  B Treatment of all people with TB, including drug-resistant TB, and patient support
  C Collaborative TB/HIV activities and management of comorbidities
  D Preventive treatment of persons at high risk and vaccination against TB
2. Bold Policies and Supportive Systems
  A Political commitment with adequate resources for TB care and prevention
  B Engagement of communities, civil society organizations, and public and private care providers
  C Universal health coverage policy and regulatory frameworks for case notification, vital registration, quality and rational use of medicines, and infection control
  D Social protection, poverty alleviation, and actions on other determinants of TB
3. Intensified Research and Innovation
  A Discovery, development, and rapid uptake of new tools, interventions, and strategies
  B Research to optimize implementation and impact and promote innovations

Here we review the current status of TB research efforts, the challenges ahead, and the way forward towards elimination, with a focus on research.

## Research Priorities in 2016

Research for TB elimination requires an intensification of efforts across a continuum from fundamental research (for improved diagnostics, treatment, and prevention), to operational and health systems research (for improved performance and introduction of new health care delivery strategies). Following the approach taken in the *International Roadmap for Tuberculosis Research* developed by WHO and the Stop TB Partnership in 2011 [4], there is a need to stimulate outcome-oriented research with a view to develop revolutionary new TB diagnostics, treatment, and prevention tools and approaches, optimize the use of existing technologies and strategies, and ensure the uptake of new TB interventions within the larger frame of system-wide health care. Research is needed in the following areas.

### Fundamental Research

Fundamental research is crucial to the development of new tools and strategies for prevention, diagnosis and cure [5]. The natural history of *Mycobacterium tuberculosis* infection in humans remains unclear [6]. Better characterization of TB pathogenesis with clear understanding of the respective contributions of the pathogen and the host is critical for the development of new tools. Development of effective new TB interventions will require detailed comprehension of the dynamic nature of the host–pathogen interaction, the phenomenon of latency (that is characterised by persistent immune response to stimulation by *M*. *tuberculosis* antigens without evidence of clinically manifested active TB [7]), and the determinants of progression from pathogen exposure to initial infection and then to the various phases of the spectrum of disease, associated with the identification of the stage-specific bacterial and host markers of this progression [8].

### Development of Diagnostic Tests

**Fig 1:**
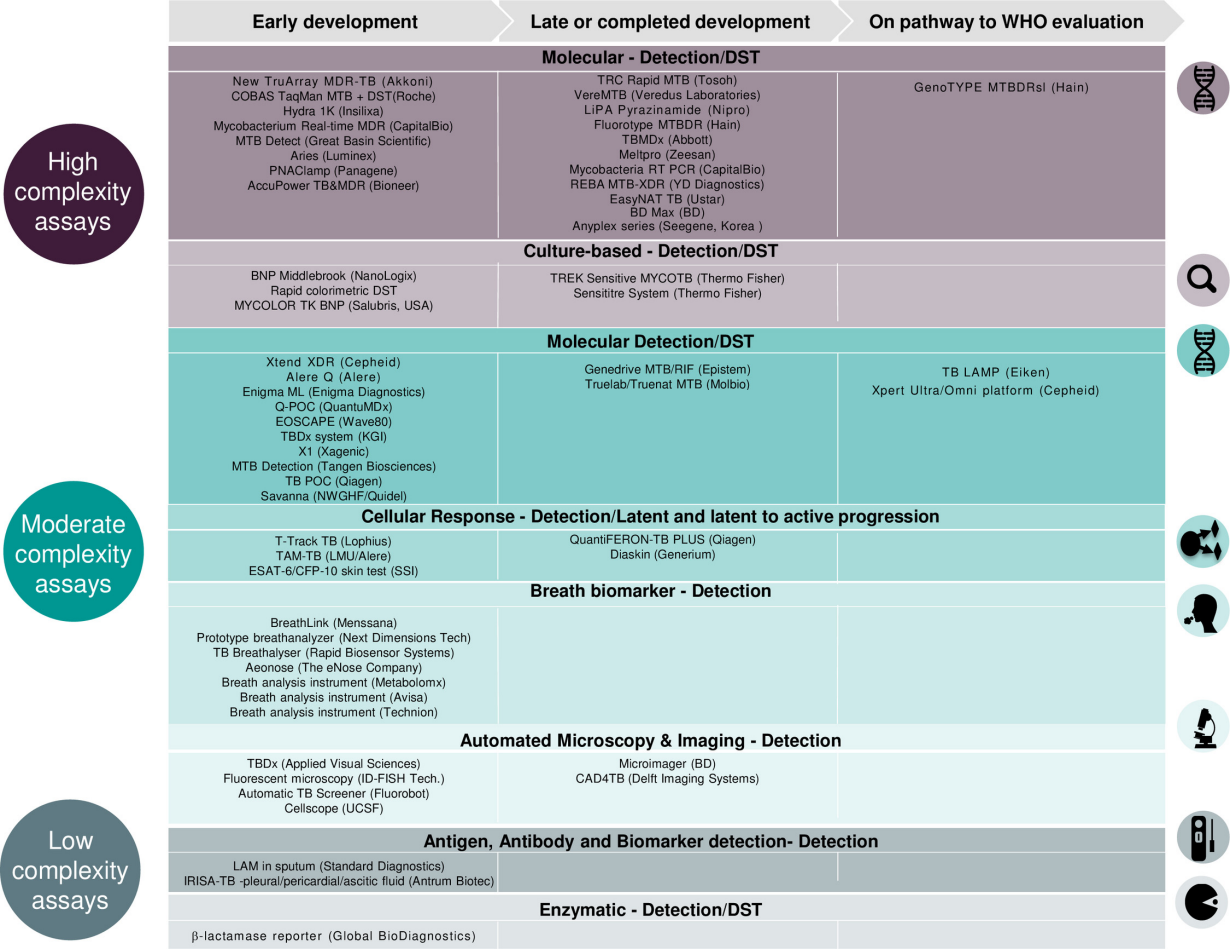
The development pipeline of new TB diagnostics. Sources: WHO Global TB Programme, 2015—http://www.who.int/tb/publications/global_report/en/; Foundation for Innovative New Diagnostics (FIND)— http://www.finddiagnostics.org/resource-centre/presentations/find_symposium_capetown_2015/index.html. Disclaimer: The mention of specific companies or of certain manufacturers’ products does not imply that they are endorsed or recommended by the World Health Organization or are preferred over others of a similar nature that are not mentioned. Errors and omissions excepted, the names of proprietary products are distinguished by initial capital letters.

There has been a proliferation of new diagnostic technologies over the past 5 years, and currently more than 50 new TB tests are in various stages of development (Fig 1) [9]. The vast majority of tests in the pipeline are still in early stages of development and/or evaluation. A few new technologies are already available on the market but have not undergone WHO evaluation yet because of limited available data. Rapid molecular diagnostics and drug susceptibility testing using automated nucleic acid amplification have been introduced but still need to be adapted for optimal use in decentralised settings [10]. Technologies under development include innovative and complex molecular platforms for simultaneous detection of multiple mutations for drug resistance [11]. Four target product profiles have been developed for high-priority tests to align the needs of end users with the specifications that product developers should meet for performance and operational characteristics of such tests [12,13]. The development of an accurate and rapid point-of-care test for TB disease, as well as a reliable test to predict the development of TB disease in latently infected persons, requires investments in biomarker research [14], as well as in transforming sophisticated laboratory technologies into robust, accurate, and affordable point-of-care platforms.

### Development of New Drugs and Regimens for the Treatment of All Forms of TB

The anti-TB drugs currently used in first-line treatments are more than 40 years old. The regimen that is currently recommended by WHO for treatment of drug-susceptible TB is highly efficacious, with cure rates of around 90% in HIV-negative patients, but is of 6 months duration and, if not taken properly, can lead to development of microbial resistance. Regimens for treatment of multidrug-resistant (MDR) TB currently recommended by WHO are lengthy (at least 20 months), associated with multiple and sometimes serious side effects, and compounded by low cure rates. There are also interactions between some anti-TB drugs and antiretroviral therapy (ART) for people living with HIV. Therefore, new drugs are required to shorten and simplify treatment, to improve the efficacy and tolerability of treatment for MDR-TB, and to improve the treatment of TB among people living with HIV.

**Fig 2:**
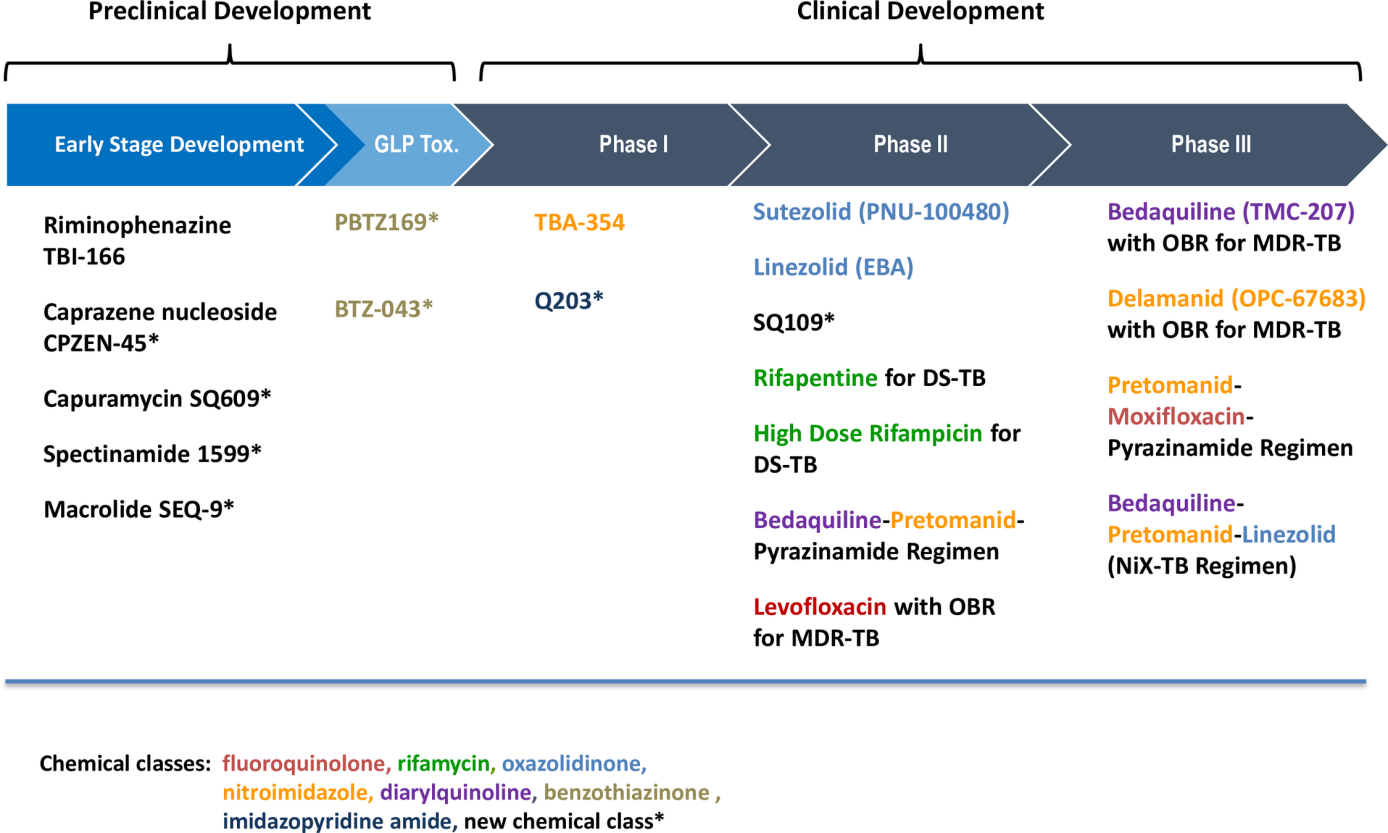
The development pipeline of new TB drugs. OBR: optimized background regimen; EBA: Early Bactericidal Activity study; DS-TB: Drug susceptible tuberculosis; MDR-TB: Multi-drug resistant tuberculosis. Sources: the Stop TB Partnership Working Group on New Drugs, 2015—www.newtbdrugs.org. Details for projects listed can be found at http://www.newtbdrugs.org/pipeline.php and ongoing projects without a lead compound series identified can be viewed at http://www.newtbdrugs.org/pipeline-discovery.php.

There are currently eight new or repurposed drugs in Phase II or Phase III trials (Fig 2). Two new drugs—bedaquiline, a diarylquinoline [15], and delamanid, a nitro-imidazole [16]—have been approved by stringent regulatory authorities (including the US Food and Drug Administration and the European Medicine Agency) and are recommended by the WHO for use in the treatment of MDR-TB under certain conditions [17,18]. Novel regimens including these and other new drugs are being studied for a shorter, safer, and simplified treatment of drug-susceptible and drug-resistant TB [19]. In parallel, novel short treatment combinations that could be safely used to prevent development of TB in latently infected persons are being tested. Development of shorter and simpler regimens combining new drugs and existing drugs requires solid information on drug–drug interactions, as well as investigation of their use in specific patient populations, such as persons with HIV/AIDS, pregnant women, and children. However, the drug pipeline appears rather static at present, with only two compounds currently in Phase I of clinical development. Major investments in drug discovery are required to expand the pipeline, facilitate the transition of promising new compounds from the preclinical to the clinical stages of development, and identify early markers of treatment outcome that would accelerate clinical development and evaluation of short regimens using novel trial designs [20]. Investments are also needed in capacity building, as well as in maintenance of trial sites, to implement trials complying with international registration standards in high-burden settings.

### Research for an Effective Vaccine against TB

**Fig 3:**
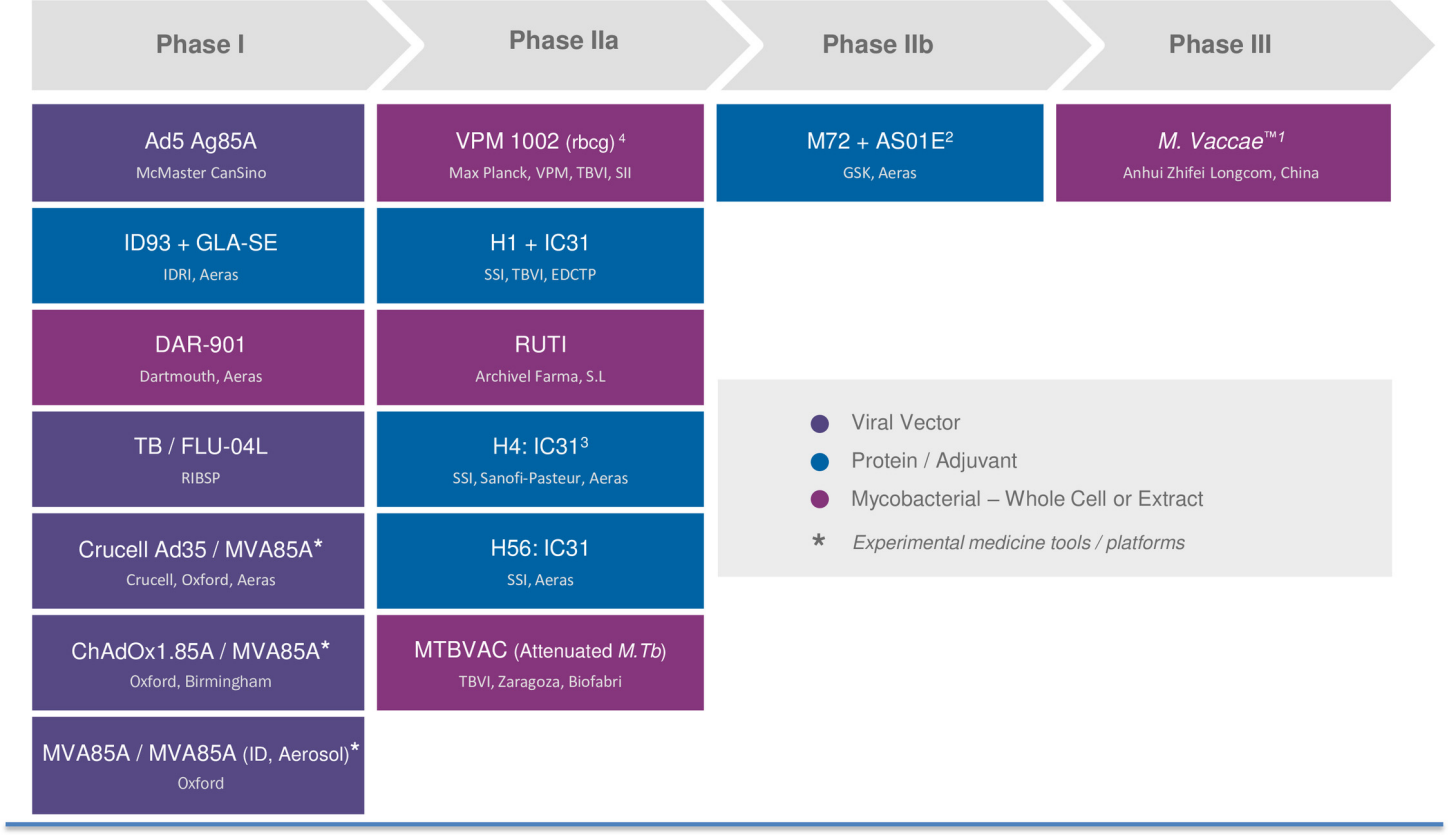
The development pipeline of TB vaccine candidates. Notes: (1) Endpoint data should be available in 2016; (2) efficacy data will likely be available in 2018; (3) prevention of infection data are likely to be available in 2017; and (4) initial safety and efficacy trials began in 2015. Sources: the Stop TB Partnership Working Group on New TB Vaccines, 2015—www.newtbvaccines.org; Aeras—www.aeras.org.

There are currently 15 vaccine candidates in clinical trials [21], including recombinant bacillus Calmette-Guérin (BCG) vaccines, attenuated *M*. *tuberculosis* strains, recombinant viral-vectored platforms, protein/adjuvant combinations, and mycobacterial extracts (Fig 3). Most candidates are designed for prevention of infection or of progression to disease in infected persons and are currently in or about to enter Phase II or IIb trials. However, there is growing concern that the strategies being pursued are too immunologically similar [22]. In the absence of known immune correlates for protective immunity against disease or infection, the portfolio must be further diversified so that candidates explore more potential immunological routes for prevention. There should be greater emphasis on early experimental trials to address basic questions about TB immune responses and vaccine delivery methods, as well as to evaluate different biologically relevant endpoints. Identification of candidate biomarkers and antigen selection and evaluation strategies are necessary to permit a more diverse pipeline [23].

### Research to Optimise Uptake and Implementation of New Tools and Interventions

Research aimed at developing interventions that result in effective policies, better design of health systems, and more efficient methods of service delivery is critical. Operational research can help assess the effectiveness of new tools or interventions and determine the conditions and requirements that will maximise their effective use [24]. Research is also needed to identify and address bottlenecks to implementation of existing policies and to provide evidence from the perspective of patients and health systems alike. On the programmatic aspect, there is a need to improve access to TB diagnostics and to improve the management of MDR-TB as well as HIV/TB coinfection and comorbidity conditions.

While accelerated TB control cannot be achieved without solid knowledge of the causative organism and its relation to humans, the social and health system context within which TB continues to flourish must also be understood [25]. Optimizing control activities at the population level requires essential understanding of the social and economic determinants of disease [26,27], the social and behavioural determinants of seeking health care, and the wider health and social system organization and dynamics in relation to TB. Research in social science as well as in health systems and policy is essential to maximize the benefits of both existing and new control strategies towards elimination.

## Challenges to Expand Research Efforts to Reach Global Targets

Greatly enhanced investments in research and development are possible through effective engagement of all stakeholders—including public and private donors, the scientific community, national TB programmes, and civil society. However, major knowledge gaps and scientific challenges remain, as shown by the disappointing results from recent vaccine and drug trials [28–31], and the capacity to address them is critically hampered by the global resource gap.

After a significant increase from about US$358 million in 2005 to about US$637 million in 2009, global funding for TB research and development (R&D) has remained static for the last 5 years [32]. The US$674 million spent in 2014 represents a financial shortfall of about US$1.3 billion per year according to the Global Plan to Stop TB 2011–2015 [33]. TB research is highly dependent on a very limited number of donors, including a few governmental agencies and philanthropic groups almost exclusively in the Organisation for Economic Co-operation and Development (OECD) countries. This creates a fragile resource base, as government donor agencies face many competing demands for funding and are subject to continuous challenges from emerging priorities. In view of the risk that available support by current major public donors may stagnate or even decline in the future and given that private pharmaceutical companies are retreating from antimicrobial R&D, particularly for TB [32], there is an urgent need to increase and diversify the investment sources and make optimal use of every dollar spent on TB research.

An additional challenge is the fragmentation of the research arena. Limited links and collaboration between entities that are involved in TB research, nationally and internationally, as well as between biomedical TB researchers and those involved in areas such as health systems, health economics, and social and behavioural research may lead to reductions in the effectiveness, depth, quality and impact of research.

Another major limitation of TB research in low- and middle-income countries (LMICs) is the lack of human capacity, i.e., an adequate number of highly trained researchers. This is a consequence of limited career opportunities and a shortage of infrastructure as well as operating funds. Research training is often inadequate, and there are limited opportunities for mentoring/support to help young researchers to become established, which may motivate young talents to go abroad. There is often an overdependency in LMICs on researchers from high-income countries to fill the training and mentoring gaps.

## The Way Forward

Enhancing research along the full spectrum and strengthening research capacity in countries with a substantial TB burden are crucial for TB elimination, so as to develop and implement widely novel strategies for optimal TB control and care. These should build on synergies between interventions on the transmission (e.g., a pre-exposure vaccine) and reactivation (e.g., a mass prophylaxis regimen and a post-exposure vaccine) pathways while ensuring rapid detection and cure of patients [34]. Recent models show that the game-changing intervention would be to act on the pool of infection with a strategy that relies on combined mass vaccination with a 60% efficacious post-exposure vaccine and preventive therapy with safe drugs and ensure that such strategies are in place from 2025 onward [35].

The challenges and opportunities to enhance research for TB elimination were discussed in a global expert consultation organised by WHO and Karolinska Institutet, Stockholm, Sweden, with support from the Swedish government, in November 2014. The consultation resulted in several concrete suggestions on the way forward.

Considering the limitations in research funding, one suggested approach is to structure large, cross-cutting research initiatives, combining basic, clinical, health system, and social science, that could be led by consortia grouping academic and other institutions at national, regional, or international levels. Such multidisciplinary, multicentre research projects, constructed around key thematic areas, could leverage existing support opportunities by a variety of funding organizations and allow synergy among different disciplines and sectors [36].

Effectively translating research into improved patient care and maximising access to products requires optimal coordination among partners, so as to undertake public health–oriented research for the development of new tools and strategies and to facilitate their seamless integration into current programmes. Mapping institutions, programmes, and individuals involved in TB research and matching these to research needs and priorities at national and international levels is fundamental to improve the relevance, quality, and efficiency of research. Coordination of research efforts could be further facilitated by the creation of networks with hubs located in research institutions with a strong capacity in a particular research area, e.g., operational [37] or vaccine research [38].

At the country level, it is essential to create a research-enabling environment that fosters and rewards high-quality research. This includes the development of country-specific, prioritized TB research agendas, inclusive of indicators to measure progress and impact. Furthermore, a broad-based, concerted effort by national governments and international donors is needed to develop research capacity at the country level [39,40]. Existing capacity-strengthening efforts, through ad hoc or institutionalized training courses, project-specific research consortia, or sandwich research training programmes should be scaled up and replicated.

Lastly, research funding should be coupled with increased partnership built on joint ownership, leadership, and trust with mutual benefit, so as to facilitate and enhance research, training, infrastructure development, and control efforts in high-burden countries [41]. Increased funding from middle-income high-TB-burden countries (especially the BRICS countries [Brazil, Russia, India, China, and South Africa]) could play a major role. At the BRICS Health Ministers’ Summit in Brazil (Nov 2014), the ministers agreed to cooperate on scientific research and innovations in TB diagnostics and treatment and to jointly identify manufacturing capacities and TB financing [42]. In addition, innovative funding mechanisms are presently being explored or implemented, and these could serve as examples of novel ways to fund R&D.

To facilitate this effort and following the recommendations arising from the Stockholm consultation, WHO has developed a Global Action Framework for TB Research to foster high-quality TB research for the next 10 years (2016–2025) at global and national levels [43]. The Framework outlines means to strengthen TB research globally, with a special emphasis on LMICs carrying the largest burden of TB. The framework has two fundamental objectives: (1) to promote, enhance, and intensify TB research and innovation at the country level through the development of country-specific TB research plans and strong research capacity, and (2) to promote, enhance, and catalyse TB research at the global level through advocacy, sharing innovations, discussion of global priorities in TB research, and development of regional and international networks for research and capacity building. It is designed for use by a wide range of groups and individuals, including ministries of health and their national TB programmes, ministries of science and technology, national research institutes, academia, researchers, international and national donors and technical agencies, nongovernmental organizations (NGOs), and civil society.

## Conclusion

The ultimate goal of translational research in TB is to improve tools and approaches for effective, diagnosis, treatment, and prevention of TB. This requires close collaboration between key stakeholders to guide development and implementation of new tools. In view of the current state of the global TB epidemic and the need for revolutionary interventions to accelerate the rate of decline of TB, research is a crucial component of the WHO End TB Strategy. Its success relies heavily on the potential to create optimal synergy among researchers and research institutions, donors, programmes, health systems, patients, and advocacy groups so that efforts are effectively carried out worldwide for enhanced, properly funded, and sustainable research for TB elimination.

## Abbreviations

ART: antiretroviral therapy
BCG: bacillus Calmette-Guérin
BRICS: Brazil, Russia, India, China, and South Africa
FIND: Foundation for Innovative New Diagnostics
LMIC: low- and middle-income country
MDR: multidrug resistant
NGO: nongovernmental organization
OECD: Organisation for Economic Co-operation and Development
R&D: research and development
TB: tuberculosis
